# Automatic Coregistration Algorithm to Remove Canopy Shaded Pixels in UAV-Borne Thermal Images to Improve the Estimation of Crop Water Stress Index of a Drip-Irrigated Cabernet Sauvignon Vineyard

**DOI:** 10.3390/s18020397

**Published:** 2018-01-30

**Authors:** Tomas Poblete, Samuel Ortega-Farías, Dongryeol Ryu

**Affiliations:** 1 Centro de Investigación y Transferencia en Riego y Agroclimatología (CITRA), Universidad de Talca, Casilla 747, Talca 3460000, Chile; totopoblete@gmail.com; 2 Research Program on Adaptation of Agriculture to Climate Change (A2C2), Universidad de Talca, Casilla 747, Talca 3460000, Chile; 3 Department of Infrastructure Engineering, The University of Melbourne, Parkville 3010, Australia; dryu@unimelb.edu.au

**Keywords:** multispectral and thermal automatic coregistration, shadow removal, crop water stress index (CWSI), UAV, midday stem water potential

## Abstract

Water stress caused by water scarcity has a negative impact on the wine industry. Several strategies have been implemented for optimizing water application in vineyards. In this regard, midday stem water potential (SWP) and thermal infrared (TIR) imaging for crop water stress index (CWSI) have been used to assess plant water stress on a vine-by-vine basis without considering the spatial variability. Unmanned Aerial Vehicle (UAV)-borne TIR images are used to assess the canopy temperature variability within vineyards that can be related to the vine water status. Nevertheless, when aerial TIR images are captured over canopy, internal shadow canopy pixels cannot be detected, leading to mixed information that negatively impacts the relationship between CWSI and SWP. This study proposes a methodology for automatic coregistration of thermal and multispectral images (ranging between 490 and 900 nm) obtained from a UAV to remove shadow canopy pixels using a modified scale invariant feature transformation (SIFT) computer vision algorithm and Kmeans++ clustering. Our results indicate that our proposed methodology improves the relationship between CWSI and SWP when shadow canopy pixels are removed from a drip-irrigated Cabernet Sauvignon vineyard. In particular, the coefficient of determination (R^2^) increased from 0.64 to 0.77. In addition, values of the root mean square error (RMSE) and standard error (SE) decreased from 0.2 to 0.1 MPa and 0.24 to 0.16 MPa, respectively. Finally, this study shows that the negative effect of shadow canopy pixels was higher in those vines with water stress compared with well-watered vines.

## 1. Introduction

Water availability is a critical limiting factor in the agricultural industry; therefore, a wide range of new technologies and strategies have been adopted to optimize the agricultural water consumption [1,2,3,4]. Granier et al. [5] argued that the measurements of physiological parameters can provide better information about the whole-plant-level water use with changing atmospheric water demands. For example, the water potential has been used to characterize the plant water stress and to schedule irrigation in vineyards [6,7,8], as well as for nuts trees [9,10], and olive trees [11,12]. However, water potential is typically measured on a plant-by-plant basis leading to high costs and requiring a considerable time when these measurements are extended to cover a large area [13,14]. This limitation has motivated the development of cost- and time-effective alternatives to evaluate plant water status.

Multispectral imagery to capture images at the leaf and canopy levels has been proposed as an effective tool for agricultural applications [15] to indirectly and remotely assess plant water status. For example, Rapaport et al. [16] reported that estimating the water balance index (WABI-2) using visible (538 nm) and short-wave infrared (1500 nm) spectrum is a good indicator of water stress in grapevines. Rallo, et al. [17] suggested that spectral information between the near infrared (NIR) (750 nm) and short-wave infrared (SWIR) (1550 nm) ranges can improve the prediction of leaf water potential. In addition, Pôças et al. in [18,19] showed that the wavelength information of visible (VIS) and NIR spectra can be used to predict water status. Poblete, et al. [20] suggested that artificial neural networks using information obtained from 500 to 800 nm could be used to predict the stem water potential (SWP) spatial variability in vineyards.

Furthermore, the Crop Water Stress Index (CWSI) derived from the radiometric temperature of a plant canopy measured using thermal infrared (TIR) sensors has been suggested as a reliable tool to assess water stress [21,22,23,24,25] showing good correlations with ground measurements of water potential. However, as in the case of ground-based water potential measurements, when large crop areas are to be assessed, the ground-based TIR measurements can still be time-consuming and impractical. Thus, remotely collected TIR imagery has been suggested as an alternative tool that can provide crop status information over large regions in a non-invasive manner [26,27,28,29]. In particular, unmanned aerial vehicles (UAV) have become a useful remote sensing tool, having significant advantages in terms of cost, versatility, and high spatial resolution [30]. The CWSI studies using UAV-borne sensors have achieved a high correlation with the plant water status measured using ground-based measurements [14,29,31,32,33].

However, the UAV-borne TIR sensing for plant water stress suffers from the technical issue of the potential degradation of the canopy temperature information by the pixels of a shaded (or shadow) canopy; this is because the surface temperature of sunlit canopy is known to better represent the plant water stress. Existing methods to remove these shaded pixels from remote-sensing images can be divided into two principal steps: shadow detection followed by a de-shadow process [34]. The first step, shadow detection, can be conducted by either thresholding or modeling [35]. The thresholding process is more common as it is less complicated than modeling, because modeling requires prior information of shadows and mathematical conceptualization; consequently, modeling is applied only to specific cases. The thresholding process involves finding the optimal threshold value of a digital number based on histograms to segregate shadow information from other types of information. Previous studies have used different wavelengths to elucidate thresholds for shadow deletion. For example, NIR (757–853 nm) [36], the ratio between blue (450–520 nm) and NIR (760–900 nm) [37], Infrared (10.4–12.5 µm) [38], and indices [39,40,41] have been used to separate undesired information. However, the TIR information obtained by the commonly used thermal imaging devices (based on an uncooled microbolometer) does not provide sufficient sensitivity for subtle temperature variation [15]; therefore, this method often fails to distinguish shadow canopy pixels from shadow soil pixels. Considering the issues with the shadow canopy pixels, an important process in thermal image processing is shadow pixel removal to improve the resampling of the sunlit canopy information [42]. Zarco-Tejada et al. [43] and Suárez et al. [44] highlight the importance of resampling sunlit canopy pixels using hyperspectral and multispectral imagery, respectively, to assess the plant water stress. Using UAV-borne thermal imagery, several studies have proposed different methodologies to achieve shadow removal and avoid the shadow effect in the case of thermal images. For example, Zarco-Tejada et al. [45] suggested that only the center portion of the canopy row be sampled to minimize the inclusion of shadow canopy pixels. Gonzalez-Dugo et al. [46] sampled the central 50% of the crown pixels of the canopy. Santesteban et al. [29] detailed the complexity of avoiding shadow information, especially in thermal imageries, and proposed a Digital Elevation Model (DEM) and Otsu [47] combined methodology to filter shadows using height differences presented in the ground.

**Figure 1:**
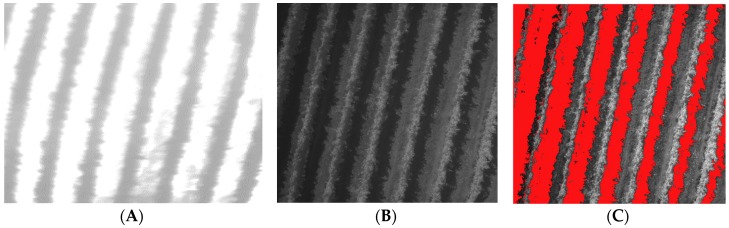
Comparison between the thermal and visible (490 nm) canopy shadow information for a drip-irrigated vineyard: (**A**) thermal image; (**B**) VIS (490 nm) image; and (**C**) VIS (490 nm) image without shadow pixels (represented in red).

Despite the proposed methodologies of shadow removal for UAV-borne images and, even if capturing images in overcast conditions can minimize the intensity of shadowing [48], the identification of shadow canopy pixels produced by the canopy over itself as information to be deleted in thermal images is not considered. For a drip-irrigated vineyard, Figure 1 shows an example of a thermal image in which the shadow canopy pixels cannot be identified on comparing with the visible imagery VIS (490 nm).

Considering the effect of shadow on water stress estimation, it is crucial to determine shaded pixels and remove them [49,50,51]. Möller et al. [23] proposed a methodology to detect grapevine crop water status using thermal and visible images collected using truck-mount sensors at 15 m above the ground to sample the sunlit canopy information; they used Ground Control Points (GCP) made of cross-marked aluminum plates to geo-reference, align, and coregister the images from two different sensors. Leinonen and Jones [42] also proposed a methodology to assess water stress in grapevine and broad bean fields using ground-obtained thermal and visible images; their methodology was based on non-automatic (by expert user) selection of GCP to overlay the images and later warp and resample the images to obtain the sunlit canopy information. Finally, Smith et al. [52] proposed a methodology to detect regions of soil moisture deficit from a spinach plantation using thermal and visible images. Bulanon et al. [53] proposed a methodology for fruit detection using thermal and VIS imagery in which four corners of a ground-marked region of interest were used to coregister VIS and TIR images and perform shadow removal. However, in all of these studies, challenges in coregistering optical and TIR images were reported when the images were combined for shadow removal [54] using non-automatic coregistration. Considering this, our study proposes an automatic scheme based on Scale Invariant Feature Transformation (SIFT) computer vision algorithm and an improved matching pairs point selection to remove shaded pixels in a UAV-borne thermal image to improve the estimation of the CWSI for a drip-irrigated Cabernet Sauvignon vineyard grown under Mediterranean climate conditions.

## 2. Materials and Methods

### 2.1. Site Description and Experimental Design

The study site has a predominant typical Mediterranean climate with a summer period from December to March that is usually dry (2.2% of annual rainfall) and hot with an average daily temperature of 21 °C, and spring that is usually wet (16% of annual rainfall). Average annual rainfall in the region is about 500 mm, which falls primarily during April to August.

Flight campaigns and climate measurements were carried out in a drip-irrigated Cabernet Sauvignon vineyard located in the Pencahue Valley, Maule Region, Chile (35°20′ L.S; 71°46′ L.W). The three-year-old wine grapes were trained on a vertical shoot positioned (VSP) system. The vineyard fractional cover, which represents the dimensionless parameter of ground covered vegetation over uncovered ground [55], was 19%. In addition, the vineyard with east–west oriented rows (at 1 m × 2 m) was irrigated daily using 2 L·h^−1^ drippers spaced at intervals of 1 m. The soil is las doscientas type with a compact arsenic soil texture with high levels of Fe and Mn.

The experimental design consisted of two completely randomized treatments (well-watered and deficit-irrigated vines) with four replications (six vines per replication). The SWP for well-watered vines showed values that ranged between −0.6 and −0.8 MPa and the deficit-irrigated vines showed values that ranged between −0.9 and −1.25 MPa. The SWP was measured at the time of UAV overflight [56] using a pressure chamber (PMS 600, PMS Instrument Company, Corvallis, OR, USA) from the middle vines for each repetition. A total of 32 leaves from the middle zone of the canopy were measured corresponding to two mature and healthy sun-exposed leaves that were previously covered with plastic bags and coated with aluminum foil for at least 1 h before measurements [6].

### 2.2. Cameras and Image Processing Description

A multispectral camera was used to collect VIS-NIR images for shadow identification. The images were obtained from a Micro MCA-6 camera (Tetracam’s Micro Camera Array), which has an array of sensors with band-path filters whose center-wavelengths are 490, 550, 680, 720, 800, and 900 nm with a resolution of 1280 (H) × 1024 (V). For thermal infrared imaging, the FLIR TAU2 640 (FLIR Systems, Inc., Wilsonville, OR, USA) was used. This camera consists of an uncooled microbolometer of 640 (H) × 512 (V) with a pixel pitch of 17 µm and spectral band ranging between 7.5 and 13.5 µm. The thermal calibration was conducted using the methodology proposed by Ribeiro-Gomes et al. [57], in which an artificial neural network is used with the sensor temperature and the digital response of the sensor as input and a Wallis filter to improve the photogrammetry process. Further, the multispectral calibration was performed using the methodology proposed by Poblete et al. [20] in which normalization of the reflectance was performed using a “white reference” Spectralon panel (Labsphere Inc., Sutton, NH, USA) and compared comparison was made with that obtained using a spectroradiometer (SVC HR-1024, Spectra Vista Cooperation, Poughkeepsie, NY, USA) to account for any relative spectral response of each band of the camera as proposed by Laliberte et al. [58].

All images from both sensors were processed using a photogrammetric software PhotoScan (Agisoft LLC, Saint Petersburg, Russia) to stitch the images together to increase the Field of View (FOV) while maintaining the intrinsic characteristics of both cameras [59]; the same software parameters proposed by Ribeiro-Gomes et al. [57] for the same type of sensor were used for stitching.

Finally, the meteorological conditions and flight description on the day of SWP are detailed in Table 1.

### 2.3. CWSI Calculation

The calculation of the CWSI was first proposed by Jones [60] and was described as follows:

(1) $$CWSI=\;\frac{T_{canopy}-\;T_{wet}}{T_{dry}-\;T_{wet}}$$

where *T_canopy_* represents the canopy temperature obtained using the UAV-borne TIR. *T_wet_* represents the temperature of a fully transpiring canopy and *T_dry_* represents the temperature of a non-transpiring fully stressed canopy. As proposed by King et al. [21] and Grant et al. [51], these values do not necessarily need to be an absolute canopy temperature limit value, but serve rather as indicator temperature to scale measured canopy temperatures to the environment for calculating relative water stress. The process for obtaining the values of *T_dry_* and *T_wet_* was based on the methodology proposed by Park et al. [31]. The process involved using an adaptive approximation based on the TIR histograms derived from the images, and then, identifying the *T_dry_* and *T_wet_* values after the shadow filtering process by considering the highest and the lowest parts of the histograms, respectively.

### 2.4. Scale Invariant Feature Transformation (SIFT) and Random Sample Consensus (RANSAC)

This algorithm was originally proposed by Lowe [61] to extract characteristic features from images in a robust manner, which is independent of variations in scaling, rotation, translations, and illumination. The algorithm workflow was summarized and explained in detail by Ghosh and Kaabouch [59]. Based on the study by Ghosh and Kaabouch [59], the five primary steps involved in this algorithm are discussed briefly in the following lines. The scale-space construction step is based on applying several Gaussian filters to the image to compute the differences between the adjacent resulting images. Then, in the scale-space extrema detection, a selection of the highest and smallest values between each point and the 26 consecutive neighbors is conducted. Further, in the keypoint localization step, low contrast and edge response points are discarded. For the resulting keypoints, the orientation assignments based on the gradient directions are computed. To define the keypoint descriptors, histograms over each keypoint orientation is calculated considering the highest peak and values under 80% as predominant directions of the local gradients.

After these five steps are performed, the nearest neighbor of a keypoint in the first image is identified from the keypoints of the second image. To remove the outliers and filtering the incorrectly matched points, the RANSAC algorithm is applied. The RANSAC algorithm was first proposed by Fischler and Bolles [62] as a resampling technique for estimating the parameters of a model, using data that may be contaminated by outliers [63]. As suggested by Derpanis [64], the RANSAC algorithm can be summarized in five principal steps: (1) randomly selectf the minimum number of points required to determine the model parameters; (2) solve for the parameters of the model; (3) determine the number of points under a tolerance value; (4) if the ratio of points resulting from the previous step over the total number of points exceeds a predefined threshold, estimate the model with a new set of points; and (5) otherwise, repeat Steps 1–4 (with a maximum of *n* iterations). Because the value selected for *n* is high to avoid mismatching, the RANSAC algorithm is time consuming [63] and has a high computational complexity when coupled with the SIFT algorithm [65]. In addition, as RANSAC is a non-deterministic algorithm [66,67], it does not guarantee the return of an optimal solution [68], resulting in different results for different runs [69]. Furthermore, when computed with few SIFT-derived keypoints, it can be sensitive to initial conditions [70]. Considering these issues, and because thermal and visible images have different characteristics with, for most cases, different spatial resolutions, their coregistering process is complex and the assumption of global statistical dependence is not completely satisfied [71]. The RANSAC algorithm between both images leads to different pairing points, which affects the overall performance and consistency in results. This statement is consistent with Turner, et al. [72], who, using RANSAC algorithm, concluded that thermal mosaics showed lower accuracy when coregistered with multispectral images, compared with visible mosaics. To address this issue, we propose an alternative filtering of matching points based on statistical parameters between previous matched pairs. Image analysis and processing were performed using the MATLAB 2017a (Mathworks Inc., Matick, MA, USA) based on the methodology proposed by Vedaldi and Fulkerson [73].

### 2.5. Slope Filtering of Matching Points

**Figure 2:**
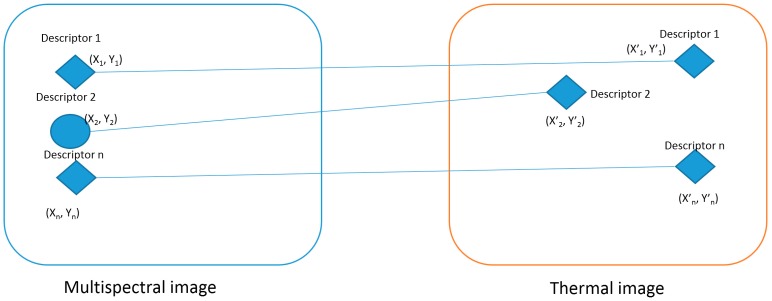
Slope calculation for previously matched descriptors points as an output of the scale invariant feature transformation (SIFT) algorithm.

As discussed above, in our method, a statistical filtering method was applied to filter previously mismatched points and our results were compared with those obtained using the RANSAC method. Our process involved both images (thermal and multispectral) as a continuous image joined by the resulting matching pair point (Figure 2).

The slope of previous matched points was calculated using the Euclidean formula as follows:

(2) $$m=\frac{\left(y_{1}^{\prime }-y_{1}\right)}{\left(x_{1}^{\prime }-\;x_{1}\right)}$$

where ($x_{1}^{\prime },y_{1}^{\prime }$) corresponds to the thermal image descriptor 1 and $\left(x_{1,}y_{1}\right)$ corresponds to the multispectral image descriptor 1.

Then, the statistical parameters were calculated for each matched feature and the filtering was conducted based on the mode of the slopes. As an example, in Figure 2, the previous matched descriptor 2 should not be considered because it was identified as a correctly matched feature, but the slope of both descriptors is different from the mode of all the slopes.

### 2.6. Shadow Filtering

As proposed by Shahtahmasseb et al. [34], histogram-based thresholding methods are commonly employed for shadow detection. In this study, with the aim of identifying the optimal wavelength for shadow detection, histograms for 112 UAV-borne images obtained from the vineyard were analyzed. The K-means clustering algorithm with k-means++ was used to optimize the thresholding [74]; this process was applied for shadow detection to six multispectral bands (490, 550, 680, 720, 800, and 900 nm) and their relative performance was compared. Five clusters and 200 iterations were selected for the classification. After performing the previous steps, the classified clusters for shadows from the six multispectral bands were used to build a mask that was applied to their RGB composition to evaluate the accuracy of the classification. Using the abovementioned process and the previously described SIFT algorithm, the resulting mask was coregistered with the thermal images to delete canopy shadow pixels.

### 2.7. Statistical Analysis

For assessing the impact of shadow canopy pixels on the linear correlation between CWSI and SWPl the coefficient of determination (R^2^) was calculated. In addition, the root mean square error (RMSE) and standard error (SE) parameters were calculated for the comparison.

## 3. Results

### 3.1. SIFT and Comparison between RANSAC and Slope Filtering for Filtering Matched Features

**Figure 3:**
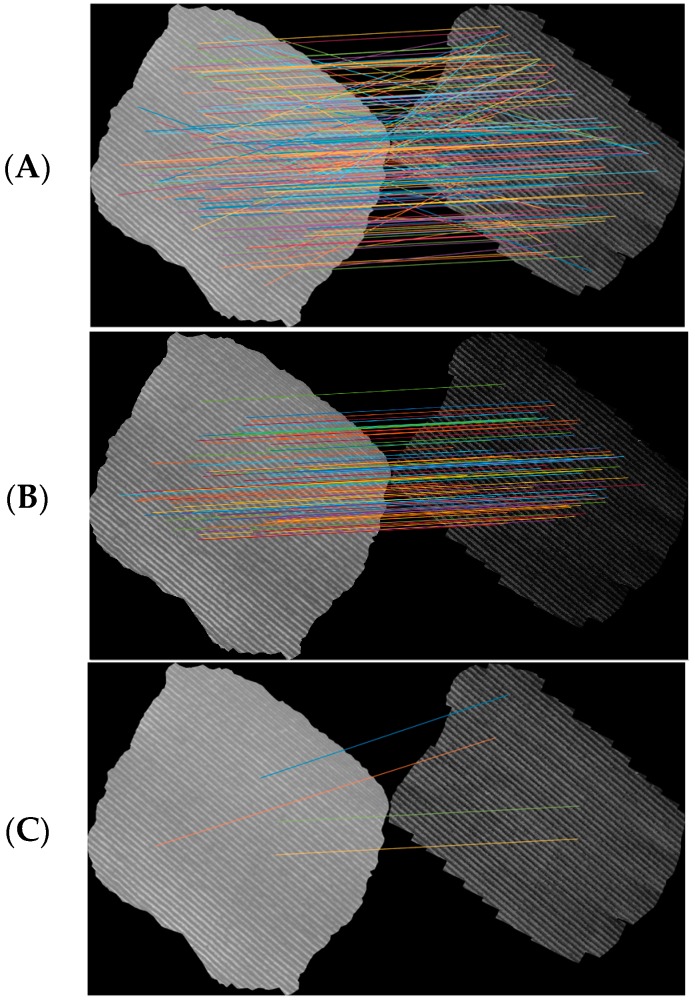
The RANSAC filtered points obtained at different times for a drip-irrigated vineyard: (**A**) initial matched points; (**B**) first execution; and (**C**) second execution.

The comparison between the abovementioned filtering processes was conducted using a complete orthomosaic obtained from the vineyard built using 112 images. The RANSAC algorithm outputs and its fluctuation on the filtered points is shown in Figure 3. Figure 3a shows the initial matched points, while Figure 3b,c shows randomly selected examples after the application of the RANSAC filter for matched points.

**Figure 4:**
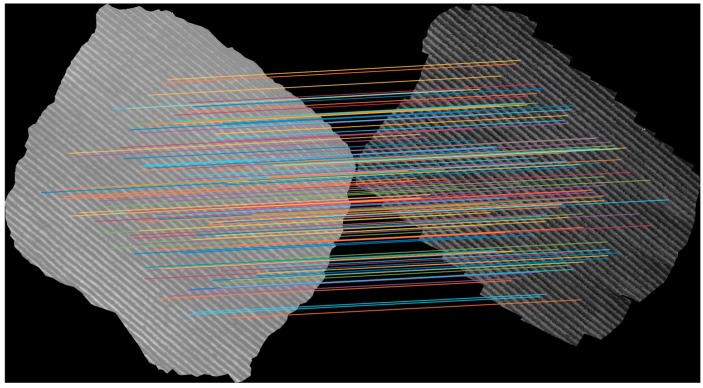
Filtered previous matched points considering the mode of the slope as a filter for a drip-irrigated vineyard.

In addition, the statistical parameters of the matching features slope are listed in Table 2. The selected statistical filter was based on the mode of the slope and its result of filtering is shown in Figure 4.

### 3.2. Shadow Filtering

#### Multispectral Band Selection for Shadow Detection

**Figure 5:**
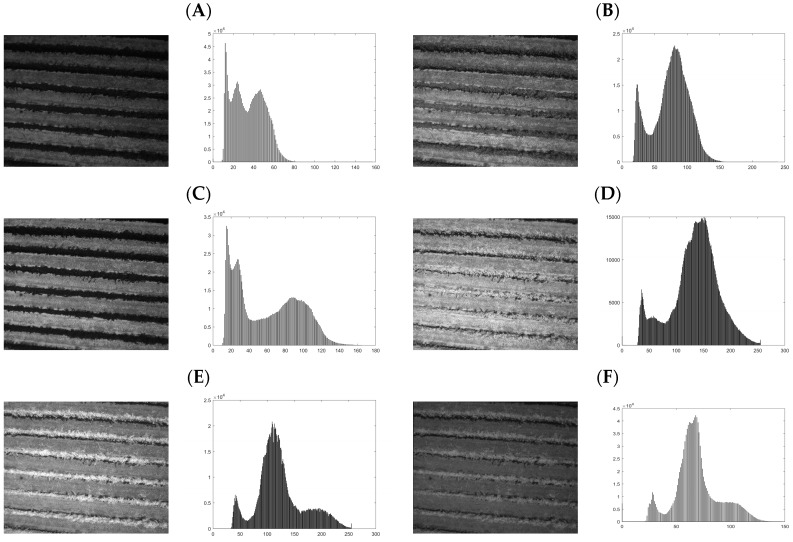
Six spectral band images and its distribution for a drip-irrigated vineyard: (**A**) 490 nm; (**B**) 550 nm; (**C**) 680 nm; (**D**) 720 nm; (**E**) 800 nm; and (**F**) 900 nm.

For shadow identification, the histogram distribution was calculated to detect peaks related to shadow information. An example of one image per band and its distribution is shown in Figure 5 for a drip-irrigated vineyard.

**Figure 6:**
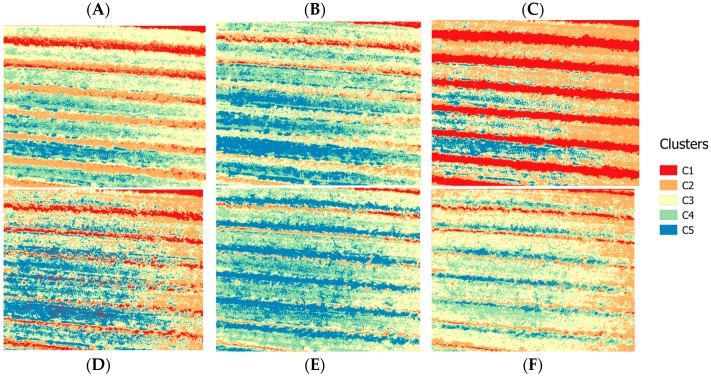
Six spectral clustered images using K-means++ algorithm for a drip-irrigated vineyard: (**A**) 490 nm; (**B**) 550 nm; (**C**) 680 nm; (**D**) 720 nm; (**E**) 800 nm; and (**F**) 900 nm.

As mentioned previously, five clusters were selected and 200 iterations were conducted for the classification of all the images. The K-means++ methodology [74] was used to set the thresholds in which shadows should be identified for the six bands. After that, each generated mask was applied to an RGB image composition to identify which better represented the shadow. Figure 6 shows the filtered images and the five identified clusters per band. As is clear in Figure 6, for each band, the clustering process allowed the identification of different types of information.

**Figure 7:**
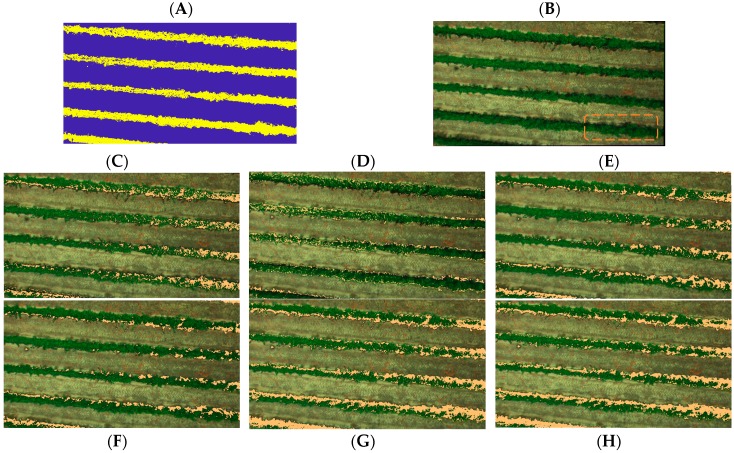
Shadow masks applied to an RGB composition for a drip-irrigated vineyard: (**A**) canopy and internal shadow mask; (**B**) RGB composition; (**C**) 490 nm; (**D**) 550 nm; (**E**) 680 nm; (**F**) 720 nm; (**G**) 800 nm; and (**H**) 900 nm.

For the 490-nm and 550-nm group of images, cluster 1 (C1) tends to identify both soil and internal shadows, while cluster 2 (C2) tends to classify vegetation information. On the other hand, for the 680-nm image, shadow is misclassified, nevertheless C1 allows directly identifying vegetation information. Finally, the 720 nm, 800 nm, and 900 nm images seem to misclassify shadow, mixing classified information in both cases with grassy soil and bare soil. To validate our method, a mask was built from the C1—680 nm image to select just vine canopy which included internal shaded canopy pixels. The resulting mask was applied over the images and K-means++ algorithm was carried out to classify vegetation and internal shaded canopy pixels (Figure 7A).

To assess and validate the accuracy of shadow identification, confusion matrices were calculated for the randomly selected marked winegrapes, as shown in Figure 7B, for the six bands to assess the percentage of correct shadow classification. The percentages of well classified shadow for 490, 550, 680, 720, 800, and 900 nm were 90%, 68%, 89%, 77%, 66%, and 58%, respectively (Table 3). Cohen’s kappa coefficient value, which is used to assess the chance-corrected agreement between two classifications [75], for each band was 0.77, 0.56, 0.76, 0.71, 0.54, and 0.41, respectively.

Based on this information, the 490-nm image, which showed the highest percentage of accuracy and Cohen’s kappa coefficient value, was selected to be coregistered with the thermal image and for thermal shadow deletion.

### 3.3. Effect of Shadow Removal on the Relationship between CWSI and SWP

To assess the impact of shadow removal on the prediction of the SWP using CWSI, UAV-borne TIR images with and without removal of shadow canopy pixels were compared.

**Figure 8:**
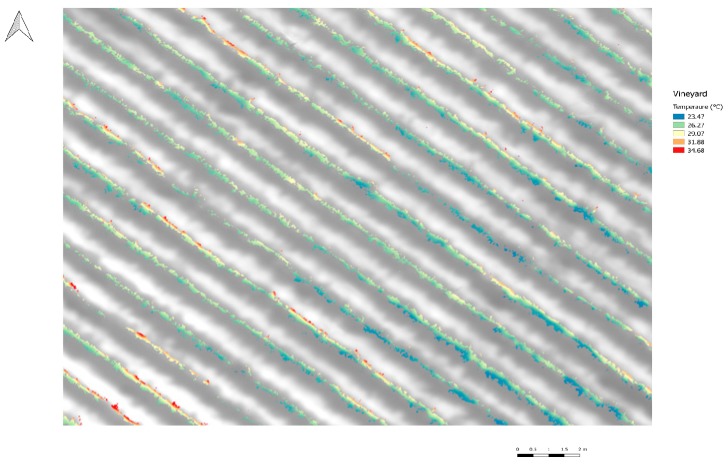
Final resulting thermal image of the drip-irrigated vineyard after automatic coregistration with the 490-nm image and filtered using the proposed shadow removal algorithm.

**Figure 9:**
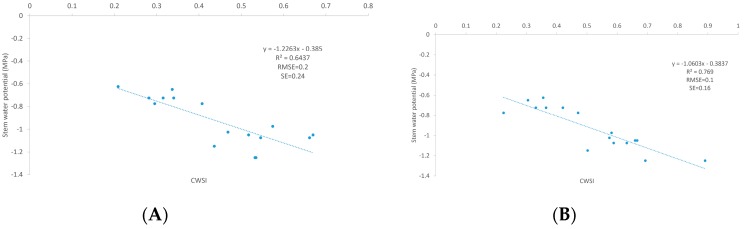
Relationships between CWSI and SWP for the vineyard: (**A**) center of the row temperature; and (**B**) temperature after coregistration with the 490-nm image and application of the proposed shadow removal algorithm.

Figure 8 shows the thermal image after automatic coregistration and shadow canopy removal. The colored regions correspond to the filtered temperature information, while the background image represents the initial vineyard information without filtered canopy shadow pixels. The mean values of canopy temperature for the cases with and without shadow canopy were 28.84 ± 1.8 °C and 29.95 ± 2.05 °C, respectively. In addition, the relationship between CWSI and SWP is shown in Figure 9. The mean values of CWSI for the non-filtered information were 0.45 ± 0.14, while those for the filtered information were 0.52 ± 0.17. Finally, the results indicated that the relationship between the CWSI and SWP improved after using the automatic coregistration algorithm. In particular, the coefficient of determination (R^2^) increased from 0.64 to 0.77. In addition, the values of RMSE and SE decreased from 0.2 to 0.1 MPa and 0.24 to 0.16 MPa, respectively.

## 4. Discussion

The selection of B1 (490 nm) as the better multispectral band for classifying shadow canopy pixels was consistent with the previous study by Ünsalan et al. [76], who used the k-means and blue information derived from the RGB spectrum to segment information avoiding shadow pixels to extract street networks and detect houses. This band selection was also proposed by Sirmacek et al. [77], who used the blue wavelength spectrum to detect shadows for building detection, suggesting that this region was dominantly better even compared with green and red for shadow pixels identification [78,79]. The selection of 490 nm image was also preferred when compared with upper wavelengths, in which blue spectrum showed better results for shadow detection increasing the performance for near infrared and shortwave infrared [80]. This validates the previous assumption that internal canopy shadow cannot be identified by TIR imagery. Considering this, the importance of coregister thermal and visible images for detecting shadow pixels was also highlighted by Leinonen et al. [42] who using ground cameras with a non-automatic methodology concluded that one of the principal steps is to correct overlapping VIS and TIR images to assess vine water status.

**Figure 10:**
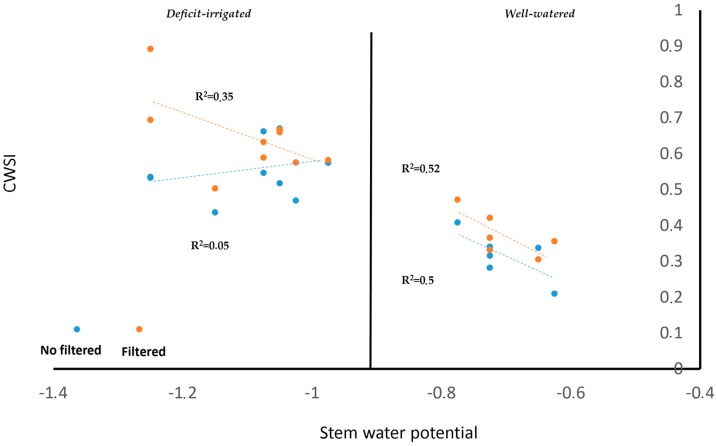
Comparison of the effect of shadow deletion on the CWSI-SWP relationship for a Cabernet Sauvignon vineyard.

In the present study, the SWP values between the stressed and well-watered vines [81] can be easily identified. The relationship between the CWSI and SWP improved when the shadow pixels were removed from the vine canopy using the suggested automatic algorithms. For the vineyard, the fractional cover was 19%, while the percentage of canopy shaded pixels was 43%. This indicates that only 8.2% of final vegetation pixels were used to develop the relationship between CWSI and SWP. Although the relationship between CWSI and SWP improved, the impact of the shadow was significant in those vines with more water stress [51]. In contrast, because no reduction of the transpiration rate occurred in well-watered vines [82], the difference between leaf temperature and air temperature was not representative [83]. These results are consistent with those of Van Zyl [49], who suggested that the impact of shadow for SWP relationships in stressed vines was considerably higher compared with sunlit leaves. In addition, Pou et al. [50] suggested that shaded canopy information negatively affects the relationship of the vine water status and CSWI because the leaf temperature decreases. Furthermore, Jones et al. [82] suggested that a greater sensitivity with respect to leaf temperature with water status measurements might be better when sunlit canopy information is considered. The effect of shadow deletion on the relationship between CWSI and SWP for stressed and well-watered vines is shown in Figure 10. Considering the results of Figure 9, in stressed vines, the shadow deletion process significantly improved the CWSI-SWP relationship with values of R^2^, increasing from 0.05 to 0.35. However, no differences were observed for well-watered vines.

## 5. Conclusions

Using a modified SIFT computer vision algorithm and Kmeans++ clustering, we performed automatic coregister UAV-TIR and UAV-VIS imagery to detect canopy shadow pixels information in thermal images. The deletion of the canopy shadow information in TIR images positively affects the relationship between the CWSI and SWP, showing an increment in R^2^ from 0.64 to 0.77. In addition, the relationship showed a decrease in RMSE from 0.2 to 0.1 MPa and in SE from 0.24 to 0.16 MPa. As future work, our methodology should be applied for validation in different cultivars, seasons, and field conditions. In addition, the impact of automatic removal of shadow canopy pixels should be assessed for evapotranspiration modeling using UAV-TIR images of vineyards.

## Figures and Tables

**Table 1:** Day of the year (DOY), Air temperature (Ta), relative humidity (RH), wind speed (u), Radiation (Rn) and phenological stage (PS) at the time of the UAV overpass during the 2016–2017 growing season; Flight and UAV description.

		**Meteorological**	**Conditions**			
**DOY**	**Flight Time (hh:mm)**	**Ta (°C)**	**RH (%)**	**u (Km/h)**	**Rn (W/m^2^)**	**PS**
6	15:00	30.81	20.2	11.3	986.7	Berry development
19	14:45	31.71	19.19	9.13	969.6	Berry development
		**Flight**	**Description**			
**Camera**	**Wavelenght**	**Resolution (pixels)**	**Altitude (m)**	**Flight Speed (m/s)**	**Overlapping (%)**	**Sidelapping (%)**
µMCA-6	490, 550, 670, 720, 800, 900 nm	1280 × 1024	30	2	90	75
Tau-2	7.5–13.5 µm	640 × 512	30	2		
			**UAV description**			
	**Model**	**Navigation controller**	**Motors model**	**Number of propellers**	**Propellers dimension**	
	Mikrokopter Okto XL	FlightNav 2.1	MK3638	8	12'' × 3.8''	

**Table 2:** Statistical parameters of slope matched points using the SIFT.

Statistical Parameter	Value
Mode	−0.3066
Mean	0.0605
Standard Deviation	0.1690
Max	1.5890
Min	−0.9278
Median	−0.0514

**Table 3:** Confusion matrix for the predicted and observed shadow information. **C1**: Shadow; **C2**: No shadow; **%**: Percentage of correctly classified shadow pixels; **Ck**: Cohen’s Kappa Coefficient.

		**Predicted**				
		**C1**	**C2**	**%**	**Ck**	
	**C1**	8220	1600	90	0.77	
	**C2**	910	11,630			**B1**
	**C1**	9090	730	68	0.56	
	**C2**	4280	8260			**B2**
	**C1**	8290	1530	89	0.76	
**Observed**	**C2**	1030	11,510			**B3**
	**C1**	9470	350	77	0.71	
	**C2**	2900	9640			**B4**
	**C1**	9630	190	66	0.54	
	**C2**	5060	7480			**B5**
	**C1**	9820	0	58	0.41	
	**C2**	7000	5540			**B6**

## References

[1] Bates B., Kundzewicz Z.W., Wu S., Palutikof J. (2008). Climate Change and Water: Technical Paper Vi.

[2] Chaves M.M., Santos T.P., Souza C.R.D., Ortuño M., Rodrigues M., Lopes C., Maroco J., Pereira J.S. (2007). Deficit irrigation in grapevine improves water-use efficiency while controlling vigour and production quality. Ann. Appl. Biol..

[3] Chapman D.M., Roby G., Ebeler S.E., Guinard J.X., Matthews M.A. (2005). Sensory attributes of cabernet sauvignon wines made from vines with different water status. Aust. J. Grape Wine Res..

[4] Berger T., Birner R., Mccarthy N., DíAz J., Wittmer H. (2007). Capturing the complexity of water uses and water users within a multi-agent framework. Water Resour. Manag..

[5] Granier C., Aguirrezabal L., Chenu K., Cookson S.J., Dauzat M., Hamard P., Thioux J.J., Rolland G., Bouchier-Combaud S., Lebaudy A. (2006). Phenopsis, an automated platform for reproducible phenotyping of plant responses to soil water deficit in arabidopsis thaliana permitted the identification of an accession with low sensitivity to soil water deficit. New Phytol..

[6] Choné X., Van Leeuwen C., Dubourdieu D., Gaudillère J.P. (2001). Stem water potential is a sensitive indicator of grapevine water status. Ann. Bot..

[7] Romero P., García J.G., Fernández-Fernández J.I., Muñoz R.G., del Amor Saavedra F., Martínez-Cutillas A. (2016). Improving berry and wine quality attributes and vineyard economic efficiency by long-term deficit irrigation practices under semiarid conditions. Sci. Hortic..

[8] Balint G., Reynolds A.G. (2017). Irrigation level and time of imposition impact vine physiology, yield components, fruit composition and wine quality of ontario chardonnay. Sci. Hortic..

[9] Nortes P., Pérez-Pastor A., Egea G., Conejero W., Domingo R. (2005). Comparison of changes in stem diameter and water potential values for detecting water stress in young almond trees. Agric. Water Manag..

[10] Espadafor M., Orgaz F., Testi L., Lorite I.J., González-Dugo V., Fereres E. (2017). Responses of transpiration and transpiration efficiency of almond trees to moderate water deficits. Sci. Hortic..

[11] Moriana A., Pérez-López D., Prieto M., Ramírez-Santa-Pau M., Pérez-Rodriguez J. (2012). Midday stem water potential as a useful tool for estimating irrigation requirements in olive trees. Agric. Water Manag..

[12] Ahumada-Orellana L.E., Ortega-Farías S., Searles P.S., Retamales J.B. (2017). Yield and water productivity responses to irrigation cut-off strategies after fruit set using stem water potential thresholds in a super-high density olive orchard. Front. Plant Sci..

[13] Acevedo-Opazo C., Tisseyre B., Guillaume S., Ojeda H. (2008). The potential of high spatial resolution information to define within-vineyard zones related to vine water status. Precis. Agric..

[14] Baluja J., Diago M.P., Balda P., Zorer R., Meggio F., Morales F., Tardaguila J. (2012). Assessment of vineyard water status variability by thermal and multispectral imagery using an unmanned aerial vehicle (uav). Irrig. Sci..

[15] Vadivambal R., Jayas D.S. (2011). Applications of thermal imaging in agriculture and food industry—A review. Food Bioprocess Technol..

[16] Rapaport T., Hochberg U., Shoshany M., Karnieli A., Rachmilevitch S. (2015). Combining leaf physiology, hyperspectral imaging and partial least squares-regression (pls-r) for grapevine water status assessment. ISPRS J. Photogramm. Remote Sens..

[17] Rallo G., Minacapilli M., Ciraolo G., Provenzano G. (2014). Detecting crop water status in mature olive groves using vegetation spectral measurements. Biosyst. Eng..

[18] Pôças I., Rodrigues A., Gonçalves S., Costa P.M., Gonçalves I., Pereira L.S., Cunha M. (2015). Predicting grapevine water status based on hyperspectral reflectance vegetation indices. Remote Sens..

[19] Pôças I., Gonçalves J., Costa P.M., Gonçalves I., Pereira L.S., Cunha M. (2017). Hyperspectral-based predictive modelling of grapevine water status in the portuguese douro wine region. Int. J. Appl. Earth Observ. Geoinf..

[20] Poblete T., Ortega-Farías S., Moreno M.A., Bardeen M. (2017). Artificial neural network to predict vine water status spatial variability using multispectral information obtained from an unmanned aerial vehicle (uav). Sensors.

[21] King B., Shellie K. (2016). Evaluation of neural network modeling to predict non-water-stressed leaf temperature in wine grape for calculation of crop water stress index. Agric. Water Manag..

[22] Gade R., Moeslund T.B. (2014). Thermal cameras and applications: A survey. Mach. Vis. Appl..

[23] Möller M., Alchanatis V., Cohen Y., Meron M., Tsipris J., Naor A., Ostrovsky V., Sprintsin M., Cohen S. (2006). Use of thermal and visible imagery for estimating crop water status of irrigated grapevine. J. Exp. Bot..

[24] DeJonge K.C., Taghvaeian S., Trout T.J., Comas L.H. (2015). Comparison of canopy temperature-based water stress indices for maize. Agric. Water Manag..

[25] Sepúlveda-Reyes D., Ingram B., Bardeen M., Zúñiga M., Ortega-Farías S., Poblete-Echeverría C. (2016). Selecting canopy zones and thresholding approaches to assess grapevine water status by using aerial and ground-based thermal imaging. Remote Sens..

[26] Zhang C., Kovacs J.M. (2012). The application of small unmanned aerial systems for precision agriculture: A review. Precis. Agric..

[27] Ortega-Farías S., Ortega-Salazar S., Poblete T., Kilic A., Allen R., Poblete-Echeverría C., Ahumada-Orellana L., Zuñiga M., Sepúlveda D. (2016). Estimation of energy balance components over a drip-irrigated olive orchard using thermal and multispectral cameras placed on a helicopter-based unmanned aerial vehicle (uav). Remote Sens..

[28] López-Granados F., Torres-Sánchez J., Serrano-Pérez A., de Castro A.I., Mesas-Carrascosa F.-J., Peña J.-M. (2016). Early season weed mapping in sunflower using uav technology: Variability of herbicide treatment maps against weed thresholds. Precis. Agric..

[29] Colomina I., Molina P. (2014). Unmanned aerial systems for photogrammetry and remote sensing: A review. ISPRS J. Photogramm. Remote Sens..

[30] Park S., Ryu D., Fuentes S., Chung H., Hernández-Montes E., O’Connell M. (2017). Adaptive estimation of crop water stress in nectarine and peach orchards using high-resolution imagery from an unmanned aerial vehicle (uav). Remote Sens..

[31] Santesteban L., Di Gennaro S., Herrero-Langreo A., Miranda C., Royo J., Matese A. (2017). High-resolution uav-based thermal imaging to estimate the instantaneous and seasonal variability of plant water status within a vineyard. Agric. Water Manag..

[32] Bellvert J., Zarco-Tejada P.J., Girona J., Fereres E. (2014). Mapping crop water stress index in a ‘pinot-noir’vineyard: Comparing ground measurements with thermal remote sensing imagery from an unmanned aerial vehicle. Precis. Agric..

[33] Bellvert J., Marsal J., Girona J., Zarco-Tejada P.J. (2015). Seasonal evolution of crop water stress index in grapevine varieties determined with high-resolution remote sensing thermal imagery. Irrig. Sci..

[34] Shahtahmassebi A., Yang N., Wang K., Moore N., Shen Z. (2013). Review of shadow detection and de-shadowing methods in remote sensing. Chin. Geogr. Sci..

[35] Liu W., Yamazaki F. (2012). Object-based shadow extraction and correction of high-resolution optical satellite images. IEEE J. Sel. Top. Appl. Earth Observ. Remote Sens..

[36] Miura H., Midorikawa S., Fujimoto K. Automated building detection from high-resolution satellite image for updating gis building inventory data. Proceedings of the 13th World Conference on Earthquake Engineering.

[37] Song M., Civco D.L. A Knowledge-Based Approach for Reducing Cloud and Shadow. Proceedings of the 2002 ASPRS-ACSM Annual Conference and FIG XXII Congress.

[38] Heiskanen J., Kajuutti K., Jackson M., Elvehøy H., Pellikka P. Assessment of glaciological parameters using landsat sat-ellite data in svartisen, northern norway. Proceedings of the EARSeL-LISSIG-Workshop Observing Our Cryosphere from Space.

[39] Hendriks J., Pellikka P. (2004). Estimation of reflectance from a glacier surface by comparing spectrometer measurements with satellite-derived reflectances. J. Glaciol..

[40] Cai D., Li M., Bao Z., Chen Z., Wei W., Zhang H. In Study on shadow detection method on high resolution remote sensing image based on his space transformation and ndvi index. Proceedings of the 18th International Conference on Geoinformatics.

[41] Sotomayor A.I.T. (2002). A Spatial Analysis of Different Forest Cover Types Using Gis and Remote Sensing Techniques.

[42] Leinonen I., Jones H.G. (2004). Combining thermal and visible imagery for estimating canopy temperature and identifying plant stress. J. Exp. Bot..

[43] Zarco-Tejada P.J., González-Dugo V., Berni J.A. (2012). Fluorescence, temperature and narrow-band indices acquired from a uav platform for water stress detection using a micro-hyperspectral imager and a thermal camera. Remote Sens. Environ..

[44] Suárez L., Zarco-Tejada P.J., González-Dugo V., Berni J., Sagardoy R., Morales F., Fereres E. (2010). Detecting water stress effects on fruit quality in orchards with time-series pri airborne imagery. Remote Sens. Environ..

[45] Zarco-Tejada P.J., González-Dugo V., Williams L., Suárez L., Berni J.A., Goldhamer D., Fereres E. (2013). A pri-based water stress index combining structural and chlorophyll effects: Assessment using diurnal narrow-band airborne imagery and the cwsi thermal index. Remote Sens. Environ..

[46] Gonzalez-Dugo V., Zarco-Tejada P., Nicolás E., Nortes P., Alarcón J., Intrigliolo D., Fereres E. (2013). Using high resolution uav thermal imagery to assess the variability in the water status of five fruit tree species within a commercial orchard. Precis. Agric..

[47] Otsu N. (1975). A threshold selection method from gray-level histograms. Automatica.

[48] Fraser R.H., Olthof I., Lantz T.C., Schmitt C. (2016). Uav photogrammetry for mapping vegetation in the low-arctic. Arct. Sci..

[49] Van Zyl J. (2017). Diurnal variation in grapevine water stress as a function of changing soil water status and meteorological conditions. S. Afr. J. Enol. Vitic..

[50] Pou A., Diago M.P., Medrano H., Baluja J., Tardaguila J. (2014). Validation of thermal indices for water status identification in grapevine. Agric. Water Manag..

[51] Grant O.M., Chaves M.M., Jones H.G. (2006). Optimizing thermal imaging as a technique for detecting stomatal closure induced by drought stress under greenhouse conditions. Physiol. Plant..

[52] Smith H.K., Clarkson G.J., Taylor G., Thompson A.J., Clarkson J., Rajpoot N.M. (2014). Automatic detection of regions in spinach canopies responding to soil moisture deficit using combined visible and thermal imagery. PLoS ONE.

[53] Bulanon D., Burks T., Alchanatis V. (2009). Image fusion of visible and thermal images for fruit detection. Biosyst. Eng..

[54] Li S., Kang X., Fang L., Hu J., Yin H. (2017). Pixel-level image fusion: A survey of the state of the art. Infor. Fusion.

[55] Morsdorf F., Kötz B., Meier E., Itten K., Allgöwer B. (2006). Estimation of lai and fractional cover from small footprint airborne laser scanning data based on gap fraction. Remote Sens. Environ..

[56] Moriana A., Fereres E. (2002). Plant indicators for scheduling irrigation of young olive trees. Irrig. Sci..

[57] Ribeiro-Gomes K., Hernández-López D., Ortega J.F., Ballesteros R., Poblete T., Moreno M.A. (2017). Uncooled thermal camera calibration and optimization of the photogrammetry process for uav applications in agriculture. Sensors.

[58] Laliberte A.S., Rango A. (2009). Texture and scale in object-based analysis of subdecimeter resolution unmanned aerial vehicle (uav) imagery. IEEE Trans. Geosci. Remote Sens..

[59] Ghosh D., Kaabouch N. (2016). A survey on image mosaicing techniques. J. Vis. Commun. Image Represent..

[60] Jones H.G. (2013). Plants and Microclimate: A Quantitative Approach to Environmental Plant Physiology.

[61] Lowe D.G. Object recognition from local scale-invariant features. Proceedings of the Seventh IEEE International Conference on Computer Vision.

[62] Fischler M.A., Bolles R.C. (1981). Random sample consensus: A paradigm for model fitting with applications to image analysis and automated cartography. Commun. ACM.

[63] Raguram R., Frahm J.-M., Pollefeys M. (2008). A comparative analysis of ransac techniques leading to adaptive real-time random sample consensus. Computer Vision–ECCV 2008.

[64] Derpanis K.G. (2010). Overview of the ransac algorithm. Image Rochester N. Y..

[65] Vourvoulakis J., Kalomiros J., Lygouras J. (2017). Fpga-based architecture of a real-time sift matcher and ransac algorithm for robotic vision applications. Multimed. Tools Appl..

[66] Michaelsen E., von Hansen W., Kirchhof M., Meidow J., Stilla U. Estimating the essential matrix: Goodsac versus ransac. Proceedings of the ISPRS Symposium on Photogrammetric Computer Vision.

[67] Meler A., Decrouez M., Crowley J.L. Betasac: A new conditional sampling for ransac. Proceedings of the British Machine Vision Conference.

[68] Bush F.N., Esposito J.M. Vision-based lane detection for an autonomous ground vehicle: A comparative field test. Proceedings of the 2010 42nd Southeastern Symposium on System Theory (SSST).

[69] Bazin J.-C., Seo Y., Pollefeys M. (2012). Globally optimal consensus set maximization through rotation search. Asian Conference on Computer Vision.

[70] Ramos F., Kadous M.W., Fox D. (2009). In Learning to associate image features with crf-matching. Experimental Robotics.

[71] Kong S.G., Heo J., Boughorbel F., Zheng Y., Abidi B.R., Koschan A., Yi M., Abidi M.A. (2007). Multiscale fusion of visible and thermal ir images for illumination-invariant face recognition. Int. J. Comput. Vis..

[72] Turner D., Lucieer A., Malenovský Z., King D.H., Robinson S.A. (2014). Spatial co-registration of ultra-high resolution visible, multispectral and thermal images acquired with a micro-uav over antarctic moss beds. Remote Sens..

[73] Vedaldi A., Fulkerson B. (2010). Vlfeat: An open and portable library of computer vision algorithms. Proceedings of the 18th ACM International Conference on Multimedia.

[74] Arthur D., Vassilvitskii S. (2007). K-means++: The advantages of careful seeding. Proceedings of the Eighteenth Annual ACM-SIAM Symposium on Discrete Algorithms.

[75] Byrt T., Bishop J., Carlin J.B. (1993). Bias, prevalence and kappa. J. Clin. Epidemiol..

[76] Ünsalan C., Boyer K.L. (2005). A system to detect houses and residential street networks in multispectral satellite images. Comput. Vis. Image Underst..

[77] Sirmacek B., Unsalan C. Building detection from aerial images using invariant color features and shadow information. Proceedings of the 23rd International Symposium on Computer and Information Sciences.

[78] Teke M., Başeski E., Ok A., Yüksel B., Şenaras Ç. (2011). Multi-spectral false color shadow detection. Photogrammetric Image Analysis, Proceedings of the ISPRS Conference, PIA 2011 Munich, Germany, 5–7 October 2011.

[79] Zhu Z., Woodcock C.E. (2012). Object-based cloud and cloud shadow detection in landsat imagery. Remote Sens. Environ..

[80] Luo Y., Trishchenko A.P., Khlopenkov K.V. (2008). Developing clear-sky, cloud and cloud shadow mask for producing clear-sky composites at 250-meter spatial resolution for the seven modis land bands over canada and north america. Remote Sens. Environ..

[81] Acevedo-Opazo C., Tisseyre B., Ojeda H., Ortega-Farias S., Guillaume S. (2008). Is it possible to assess the spatial variability of vine water status?. OENO ONE.

[82] Jones H.G., Stoll M., Santos T., Sousa C.d., Chaves M.M., Grant O.M. (2002). Use of infrared thermography for monitoring stomatal closure in the field: Application to grapevine. J. Exp. Bot..

[83] Idso S.B. (1982). Non-water-stressed baselines: A key to measuring and interpreting plant water stress. Agric. Meteorol..

